# Covalent Epitope Decoration of Carbon Electrodes using Solid Phase Peptide Synthesis

**DOI:** 10.1038/s41598-019-54000-9

**Published:** 2019-11-28

**Authors:** Lindsay Candelaria, Peter N. Kalugin, Brian M. Kowalski, Nikolai G. Kalugin

**Affiliations:** 1 Department of Materials and Metallurgical Engineering, New Mexico Tech, 801 Leroy Pl., Socorro, NM 87801 USA; 2 Harvard Medical School, 25 Shattuck Street, Boston, MA 02115 USA

**Keywords:** Extracellular recording, Sensors and biosensors

## Abstract

Long-term, minimally perturbative brain electrophysiology requires electrodes to seamlessly integrate into surrounding tissue. In this work, we demonstrate electrodes composed of covalently functionalized graphite, decorated with various functional affinity and epitope tags, and use them to detect changes in electrical potential on the surfaces of illuminated quantum dots and near fluorescing molecules. Affinity and epitope tagging of carbon was achieved using direct attachment of biotin and solid phase peptide synthesis (SPPS) of histidine (His)- and human influenza hemagglutinin (HA)-tags. Surface modification was confirmed with Auger, Energy-Dispersive X-ray (EDX), Raman, and fluorescence spectroscopy. Photoresponse was detected with compatible binding protein-surface tag combinations, confirming desired tag and electrode functionality. These results provide a path to organic, biofunctionalized, fully molecularly-defined electrodes for neuronal applications, and to a wide range of other secondary reactions and modifications of carbon; potential uses include affinity chromatography, DNA sequencing technologies, biomolecular sensors, and surfaces and scaffolds for targeted interfaces with biological tissues.

## Introduction

Since Hodgkin and Huxley’s studies with the intracellular microelectrode, a large arsenal of electrophysiologic tools has been developed to measure and alter membrane voltage in cells^1^. In neuroscience in particular, extracellular microelectrode arrays (MEAs) and intracellular recordings using patch clamp or cell-penetrating electrodes remain the gold standard for such studies. These approaches have met a number of challenges related to signal strength and quality, robustness of connections over time, and scalability to interacting with many cells at once, not to mention their physically invasive nature^2–4^. Advances in molecular biology have ushered in the era of all-optical electrophysiology, but these techniques invariably rely on dyes or transgenic fluorescent proteins, limiting their clinical applications^5^. There remains a clear need for improved electrode-based technologies to interact with the brain.

Recent developments in materials science have made significant strides toward overcoming some of the limitations of traditional electrophysiology. MEAs of a wide range of designs have been devised, with high sensing areas and electrode densities for large-scale, long-term measurements of extracellular potentials^6^. Microfabrication of electrode surfaces into various geometries, such as nanowires and mushrooms, has allowed for the detection of finer signals, and cell membrane incorporation of electrodes through endocytosis or electroporation has resulted in intracellular-like recordings from MEAs^7^. Incorporation of nonmetallic materials such as silicon, carbon nanotubes (CNTs), and different types of conductive polymers has further diversified the field, providing finer control of the biophysical and electronic parameters of the cell-electrode interface, reducing toxicity and scarring, and bringing semiconductor field-effect transistors directly to the cell surface^8–13^. Integration of electrodes into flexible polymer substrates has introduced mesh electronics, which promises unprecedented control of MEA conformation to brain anatomy, and thus improved long-term recordings due to reduced tissue perturbation and damage^14–16^. However, these technologies are still in their nascent stages, and much remains to be understood about the long-term effects of electrodes on neuronal viability and function.

One material that has emerged as a particularly attractive choice for biocompatible electrodes is carbon, thanks to its structural stability and highly tunable electronic properties. Aside from CNTs, which were primarily used for the modification of electrode surface roughness to alter cell adhesion and impedance, other forms of carbon such as graphite and graphene have also found applications^17,18^. These allotropes have improved conductivity compared to CNTs, and are especially amenable to fine control of their physical and chemical features through a variety of methods; for instance, flexible MEAs with graphitic electrodes have recently been created using laser lithography^19^. Most importantly, a number of breakthroughs have finally opened these materials up to the full spectrum of organic chemistry. Covalent functionalization of graphite and graphene was demonstrated, for example, using the Diels-Alder reactions with tetracyanoethylene, maleic anhydride, 9-methylanthracene, and 2,3-dimethoxy-1,3-butadiene^20^. More recently, covalent functionalization of pristine graphene and graphite at both edge and internal molecular sites has been achieved with benzyne^21^ and tetracyanoethylene oxide (TCNEO)^22^, the latter of which decorated the carbon surface with reactive nitrile groups. Subsequent attachment of more complex functional groups resulted in a material that could be used as a stationary phase for chiral chromatographic separation^23^.

With TCNEO-modified graphene and graphite established as versatile substrates for organic reactions, we hypothesized that they could in the same way be made selective in adhesion to particular biological structures based on surface chemistry. Additionally, we looked to leverage the intrinsic electronic capabilities of these materials and utilize them to detect local fluctuations in electrical potential, with the eventual intention of using them to interface with neurons in the brain in a surface-specific manner. We simulated these desired functions using quantum dots (QDs) coated with specifically adhesive proteins. This allowed us to both confirm attachment selectivity as well as to induce minute voltage changes directly at the electrode surface by optically exciting and polarizing the attached quantum dots.

In this work, we demonstrate electrodes composed of TCNEO-modified graphite, decorated with various functional affinity and epitope tags, and successfully implement them to detect changes in electrical potential on the surfaces of illuminated quantum dots and near fluorescing molecules^24^. Affinity and epitope tagging was achieved using direct attachment of biotin and solid phase peptide synthesis (SPPS) of histidine (His)- and human influenza hemagglutinin (HA)-tags, and surface modification was confirmed with Auger, Energy-Dispersive X-ray (EDX), Raman, and fluorescence spectroscopy^25^. Photoresponse was detected with compatible binding protein-surface tag combinations, confirming desired tag and electrode functionality. These results are promising steps on the path to organic, biofunctionalized, fully molecularly-defined electrodes for neuronal applications, with potential to address some of the fundamental problems of electrophysiology related to tissue disruption and toxicity, stability of long-term contacts, and signal quality and specificity.

## Results

### Spectroscopic characterization of graphite surface modifications

**Figure 1:**
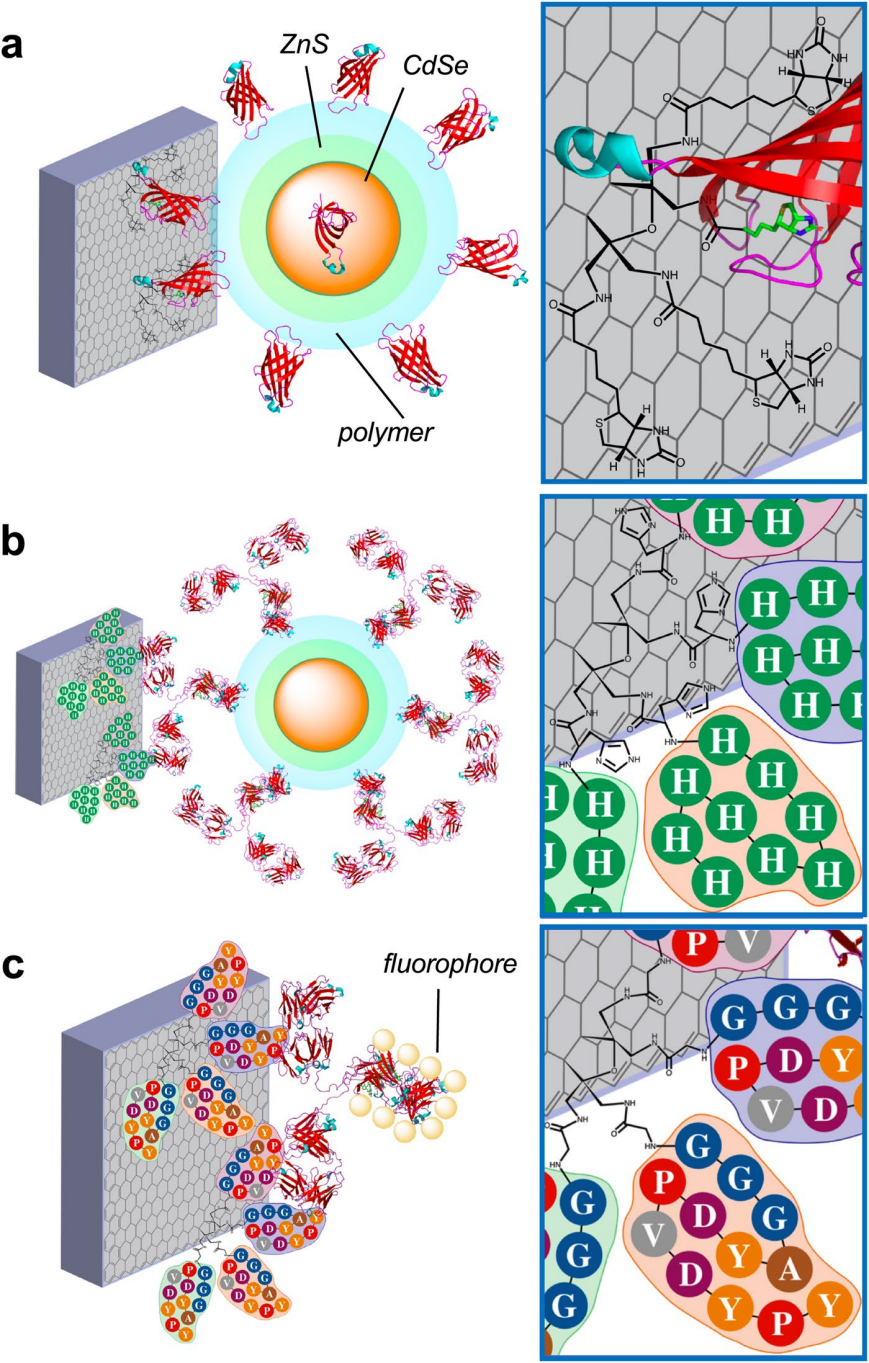
Surface-tagged graphite surfaces with attached biomolecules. (**a**) Biotin-functionalized surface with associated streptavidin-coated quantum dot. (**b**) His-tag-functionalized surface with associated His-tag antibody-coated quantum dot. (**c**) HA-tag-functionalized surface with associated fluorophore-coated HA-tag antibody. Insets show molecular detail of graphite surface attachments. Some amino acids are drawn as spheres with corresponding letter codes for simplicity of view (H = histidine, G = glycine, A = alanine, Y = tyrosine, D = aspartic acid, P = proline, V = valine). Streptavidin crystal structure obtained from PDB 1KTR, and IgG crystal structure obtained from PDB 1IGT.

Surface-tagged carbon electrodes were synthesized via TCNEO modification of bulk graphite, reduction, and direct biotinylation or SPPS attachment of His- and HA-tags, as described in Methods (Fig. 1). Changes in graphite surface chemistry were tracked between modification stages using Auger, Raman, and EDX spectroscopy, as well as Scanning Electron Microscopy (SEM).

Auger spectra^26^ of graphite samples faithfully reflected every stage of modification, with the presence and intensity of peaks from different elements corresponding to each chemical reaction (see Methods for details). For instance, Auger spectra of TCNEO-treated graphite show the clear appearance of the nitrogen peak, which is then significantly dampened along with a concomitant appearance of the oxygen peak following reduction (Fig. S1a, see Supplementary Information). This is consistent with removal of nitrogen upon rapid oxidation of the reduced material in ambient air during sample loading into the spectrometer. After biotinylation, there are expected changes in the intensities of carbon, nitrogen, and oxygen peaks, as well as the appearance of a sulfur peak (biotin composition is C_10_H_16_N_2_O_3_S) (Fig. S1b). No substantial changes in carbon, nitrogen, oxygen, and hydrogen peaks were noted across the SPPS steps, as all amino acids used in fabrication consisted only of these elements (Fig. S1d,e). Finally, following the later addition of quantum dots, Zn and Cd peaks appeared, and the S peak intensified, in line with quantum dot composition (CdSe spheres with ZnS shells and polymer coating) (Fig. S1c–e). Notably, no contaminating peaks from residual reactants were observed.

Raman spectra also varied significantly across the fabrication steps. Untreated graphite showed a characteristic Raman signature of major lines: the D-line at 1330–1350 cm^−1^, the G-line at 1582 cm^−1^, the 2D line at around 2700 cm^−1^, and a broad-range background (Fig. S2a)^27^. The relatively strong D-line, as well as the presence of spectral background, indicate the rather non-uniform structure of the utilized material. TCNEO-treated graphite demonstrated significant spectral changes, with the appearance of a strong broad-range fluorescent signal, consistent with prior findings^22,23^. Upon reduction, the fluorescence intensity decreased, consistent with the Auger results above. The C-N line at around 2230 cm^−1^, usually appearing in Raman spectra of TCNEO-modified few-layer graphene or highly uniform graphite, was masked by strong fluorescence signal in our samples^23^. Biotinylation and SPPS induced further changes in the spectra, particularly in the fluorescence component. For instance, biotinylation produced a fluorescent signal at least an order of magnitude greater in intensity than that in reduced TCNEO-modified graphite (Fig. S2b). Both His- and HA-tag synthesis resulted in wide fluctuations in fluorescence intensity, with signals increasing, decreasing, and increasing again as the peptide chains lengthened (Fig. S2c–f). This may be dependent on steric interactions between amino acid side chains, reflecting configuration changes as the peptides grow and fold into their secondary structures.

EDX spectra of the samples were not as sensitive as Auger and Raman for sequential changes due to the surface reactions. However, the spectra did indirectly report on these chemical treatments by detecting residual reactants. For instance, EDX spectra^26^ of TCNEO-treated graphite showed not only the expected signals from carbon (C K-line at 0.246 keV), nitrogen (N K-line at 0.384 keV), and oxygen (O K-line at 0.515 keV), but also a chlorine line (Cl K-line at 2.691 keV), reflecting residual chlorobenzene from the reaction (Fig. S3a). Similarly, the EDX spectra of reduced TCNEO-treated graphite showed signal from Al (Al K-line at 1.486 keV), derived from the LiAlH_4_ reactant (Fig. S3b). Spectra from further reaction and modification steps demonstrated lines from Na, P, K, and Cl following phosphate-buffered saline (PBS, a mixture of NaCl, KCl, Na_2_HPO_4_, and KH_2_PO_4_), bovine serum albumin (BSA), and CH_2_Cl_2_ treatment, F following trifluoroacetic acid (TFA) treatment, and S and Si following the later addition of quantum dots, with Si presumably present in the QD polymer coating (Fig. S3c–j)^28^. Superposition of EDX spectra of reactant-derived elements onto SEM images of samples shows a predilection of these signals for defects and cracks in the graphite, suggesting the trapping of reactants in particularly sterically restricted areas of the samples (Fig. S4).

### Confirmation of functional surface tag decoration

**Figure 2:**
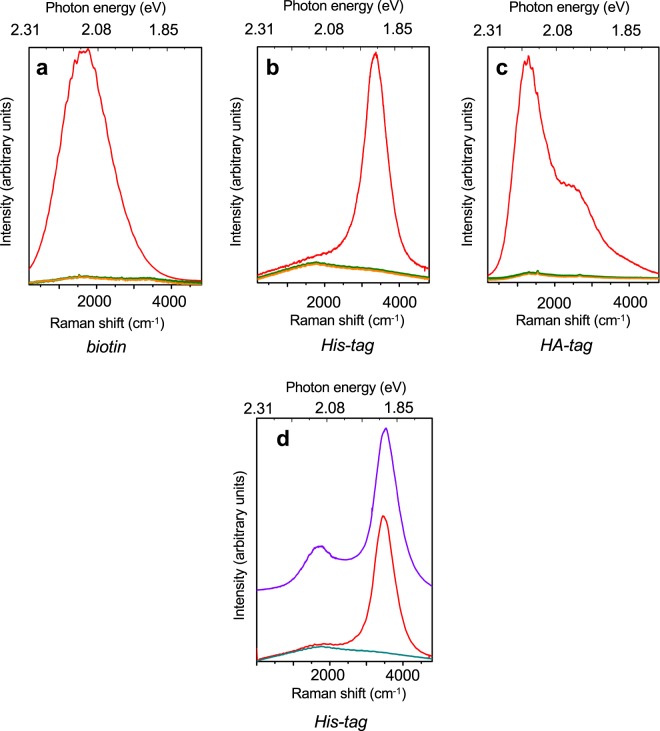
Selective attachment to surface-tagged graphite surfaces. (**a**) Raman and fluorescence spectra of biotin-functionalized graphite surfaces prior to binding protein treatment (orange), after incubation with streptavidin-coated quantum dots (red), and after incubation with His-tag antibody-coated quantum dots (olive). (**b**) Raman and fluorescence spectra of His-tag-functionalized surfaces prior to binding protein treatment (orange), after incubation with His-tag antibody-coated quantum dots (red), and after incubation with streptavidin-coated quantum dots (olive). (**c**) Raman and fluorescence spectra of HA-tag-functionalized surfaces prior to binding protein treatment (orange), after incubation with fluorophore-coated HA-tag antibodies (red), and after incubation with His-tag antibody-coated quantum dots (olive). Note the robust signals in cases of correctly paired surface tags and binding proteins, and absence of signal in mismatched samples. (**d**) Label retention on His-tag-functionalized surface. Purple line shows the fluorescence spectrum of a mixed solution of 1:10 dilution streptavidin-coated quantum dots (585 nm, 2.12 eV) and 1:10 dilution His-tag antibody-coated quantum dots (655 nm, 1.89 eV). Upon incubation of mixture on His-tag-functionalized surface and repeated washing, only the 1.89 eV fluorescence peak corresponding to His-tag antibody-coated quantum dots remains (red). For comparison, Raman/fluorescence spectrum of bare His-tag-functionalized graphite surface is also shown (olive). Peak energies in Raman shift (cm^−1^) and photon energy are shown for clarity. Excitation wavelength 532 nm. The corresponding type of graphite functionalization is indicated in *italics*.

To confirm the functionality of surface-attached biotin and epitope tags, differential affinity experiments were performed using streptavidin (Qdot 585, 585 nm/2.119 eV emission)- and anti-His-tag antibody (WesternDot 655, 655 nm/1.893 eV emission)-coated CdSe quantum dots in ZnS shells^29^, as well as fluorophore-labeled anti-HA-tag antibodies (DyLight 550, 576 nm/2.152 eV emission). First, surface-tagged graphite samples were incubated with solutions of individual labels and assessed for retention of appropriate binders after washing by measurement of their fluorescence/Raman spectra. As expected, biotinylated graphite retained streptavidin QDs but not anti-His-tag QDs, His-tagged graphite retained anti-His-tag QDs but not streptavidin QDs, and HA-tagged graphite retained anti-HA-tag fluorophores but not anti-His-tag QDs (Fig. 2a–c). Notably, while the QDs exhibited symmetrical single emission peaks at their predicted wavelengths, the DyLight fluorophore had an additional minor peak around 615 nm/2.02 eV, consistent with its vendor-provided spectrum. In all cases, retention of the incompatible label was minimal, as evidenced by the background-level fluorescent spectra of such samples.

To further examine the excellent selectivity of surface-tagged graphite, a mixed-label experiment was also performed. In this case, a mixture of streptavidin QDs and anti-His-tag QDs was applied to His-tagged graphite. While the mixed solution of QDs exhibited both the 585 nm and 655 nm emission peaks of its components, only the 655 nm peak of anti-His-tag QDs appeared on the graphite following incubation and washing (Fig. 2d). Surface-tagged graphite surfaces thus have strong and specific affinity for matching binding proteins.

### Electrode functionality and photoresponse of surface-tagged graphite

**Figure 3:**
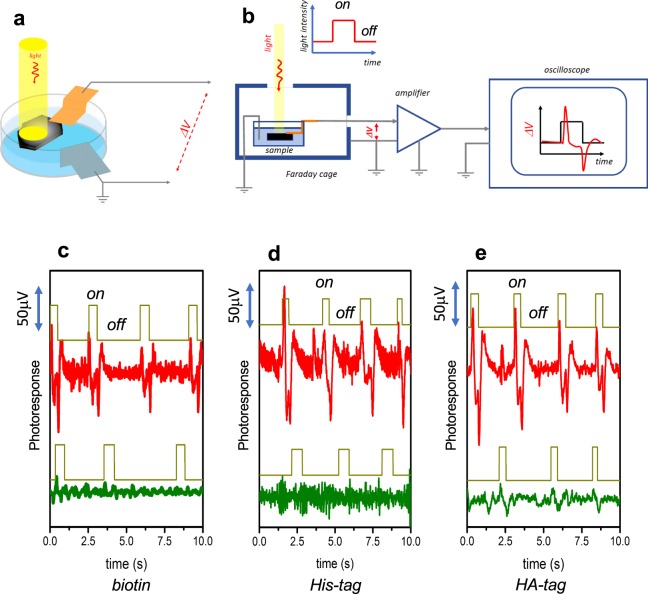
Detection of photoinduced electrical potentials near quantum dot surfaces and chemical fluorophores. (**a**) Cuvette filled with deionized water, containing the labeled surface-tagged graphite and leads connected to the sample and the water. Light path restricted by Faraday cage is shown. (**b**) Schematic of the full electronic setup, including sample and leads in Faraday cage, amplifier, and oscilloscope. As shown, light is delivered as a pulse of uniform intensity, and the oscilloscope returns peaks at times when a voltage is induced and current flows, i.e. when light intensity changes. Signals were amplified with gain of 1000–2500. Photoinduced electrical potential traces were obtained with irradiation by a broad-spectrum quartz-halogen lamp for (**c**) biotin-functionalized graphite incubated with streptavidin-coated quantum dots (red) and His-tag antibody-coated quantum dots (olive), (**d**) His-tag-functionalized graphite incubated with His-tag antibody-coated quantum dots (red) and streptavidin-coated quantum dots (olive), and (**e**) HA-tag-functionalized graphite incubated with fluorophore-coated HA-tag antibodies (red) and His-tag antibody-coated quantum dots (olive). Photoresponse is evident when binders and surface modifications are paired correctly, while minimal to no signal is detected in mismatched surface-binder pairs. The corresponding type of graphite functionalization is indicated in *italics*.

Electrophysiologic measurements require signal acquisition systems that are sensitive enough to detect minute changes in electric fields near their surfaces with appropriate temporal resolution. To simulate local voltage fluctuations, we used surface-functionalized graphite complexed with the same fluorescent binders as above. Selectively-labeled surface-tagged graphite samples were placed into a cuvette filled with deionized (DI) water, with leads connected to an amplifier and oscilloscope, protected from external electromagnetic noise by a Faraday cage (Fig. 3a,b). The cage had a small window to allow external illumination of the sample surface, with the leads outside the light path. Samples were not biased, and the photovoltaic signal between the leads induced by light illumination of the samples was amplified with a gain of 1000–2500. Samples were illuminated with flashes of 1–3 seconds in length.

Photoresponse was first assessed using a bright, broad-spectrum quartz-halogen lamp. Strong signals well above the noise level, in the tens of microvolts, were observed in samples incubated with fluorescent labels corresponding to the surface modifications (Figs. 3c–e, S5a–c). On the other hand, samples incubated with incompatible labels exhibited either undetectable or very weak (on the order of several microvolts) signals. The photoresponse of biotinylated and HA-tagged graphite in the absence of associated bright sources of fluorescence may reflect intrinsic photosensitivity of the attached molecules, in line with the strong fluorescence components in the Raman spectra of these materials. To further characterize the observed signal characteristics, the experiments were repeated using illumination with laser sources emitting at 532 nm, 633 nm, and 785 nm. As expected, photoresponse demonstrated wavelength dependence, confirming its reliance on the specific attachment of fluorescence sources (Fig. S5d–f). Biotinylated graphite with streptavidin QDs exhibited strong photoresponse to 532 nm excitation, above the QD emission wavelength of 585 nm, but no detectable signal with 633 nm excitation. His-tagged graphite with anti-His-tag QDs had strong photoresponse to 633 nm excitation, above the QD emission wavelength of 655 nm, but no response to 785 nm excitation. HA-tagged graphite with anti-HA-tag fluorophores also gave strong signal with 532 nm excitation, above the 576 nm fluorophore emission peak, but did not respond to 785 nm excitation.

**Figure 4:**
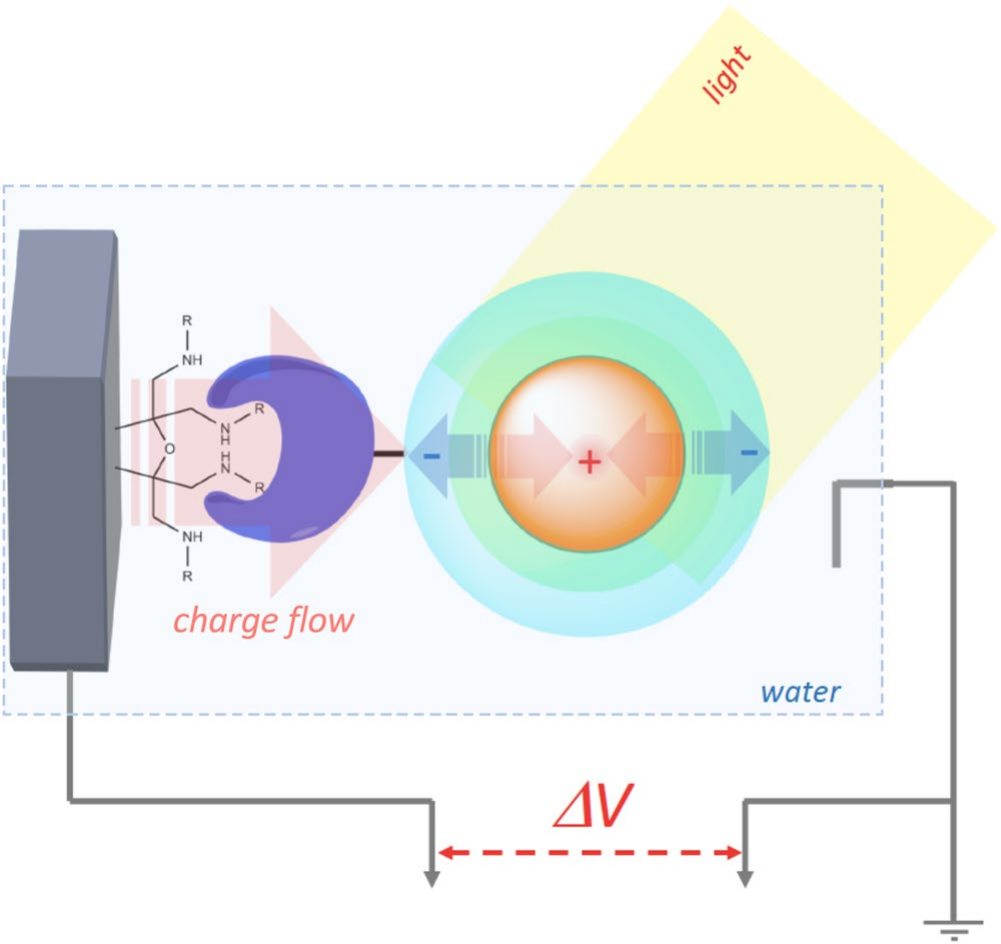
Proposed model of quantum dot surface potential generation. Optical excitation of quantum dot results in asymmetric charge distribution, with positive charge redistributed towards the center of the quantum dot and increased negative charge on the surface. This induces polarization in the linker molecules and graphite surface, with positive charge flow toward the quantum dot, resulting in measurable transient potential difference between graphite electrode and ground. Deexcitation of quantum dot restores original charge distribution, leading to positive charge flow away from the quantum dot and a transient potential difference in the reverse direction.

The photoresponse signals in all above experiments had a characteristic shape, with a transient deflection in the voltage at the start of the light pulse and a symmetrical opposite deflection when the light was extinguished. These voltage “spikes” are analogous to those previously measured in QD-decorated conductive polymers, and reflect intrinsic properties of QDs^30^. It is known that CdSe/ZnS crystals have different geometries for electron and hole wave functions, and the electron states in QDs are significantly asymmetric^29,31^. Optical excitation of QDs therefore leads to asymmetric redistribution of charge, with a more symmetric cloud of holes localized at the QD center and a less localized and more asymmetric cloud of electrons extending its wave functions outside the QD shell, causing an electric potential to appear on the QD surface (Fig. 4). The corresponding photogenerated electric field redistributes electrons in the proximal bridging molecules and the graphite itself, inducing a current in the material and a corresponding potential difference between the graphite and the ground potential of the DI water bath. Once the QD-derived field is balanced by a commensurate charge asymmetry in the graphite, the current stops and the potential difference disappears. Extinguishing the light and allowing the QDs to deexcite then triggers a current in the opposite direction.

## Discussion

We have demonstrated functional graphitic electrodes covalently decorated with bioactive surface tags. These electrodes selectively attach to matching binding proteins, and using these electrodes, one can detect microvolt-range fluctuations in local electrical potentials. QDs and fluorophores are not ideal models of neuronal behavior, and their surface electrical potential fluctuations do not reflect the dynamics of transmembrane ion movement. However, the sufficiently low level of noise from the carbon electrodes allowing for the measurement of such fine voltages is promising for their potential future use as extracellular microelectrodes, following microfabrication to appropriate cellular dimensions, which can be achieved using standard methods^7^. Notably, the surface modifications are fully molecularly-defined and chemically robust, remaining stable and functional through repeated reaction cycles, and the graphite retains its electronic properties following their attachment. This is a significant advancement over previous graphite- and pristine graphene-based biointerfaces and electrodes, which either left the material completely bare or coated it with a weakly adsorbed layer of peptides or proteins such as poly-lysine or laminin^18,32,33^. Due to its inert chemistry, carbon is often cited as an ideal material for biological interfaces. However, while it is true that carbon is likely less immunogenic than many metals due to its relative chemical inertness, it nevertheless exposes the body to foreign molecular structures and epitopes, and as such has the potential to induce an immune response^34^. By achieving precise molecular control of the carbon surface through covalent modification and decorating it with epitopes known to be non-immunogenic, it may be possible to fully encapsulate the carbon and further reduce potential immune responses.

One longstanding concern in the field of extracellular microelectrodes is electrode-cell coupling^7^. In particular, the interface between an extracellular electrode and the cell surface consists of a gel-like matrix containing water and ions as well as extracellular proteins, polysaccharides, lipids, and other molecules. The classical interpretation of this interface treats the ionic fluid as the primary conductor passing current between the cell and electrode, equalizing their electrical potentials. An ideal electrode should be decoupled from this extracellular conducting fluid to avoid ionic shielding and minimize stray current flow. In the case of our affinity-modified electrodes, such decoupling may be achievable both by tight binding to the cell surface via antibodies or other proteins or lipids, maintaining a consistent cell surface-electrode distance, as well as steric displacement of intervening water and ions through dense covalent functionalization of the electrode surface. Importantly, coupling between the signal source and electrode can be DC or AC, and mediated not only through the water bath solution but also the connecting molecular chain of streptavidin/antibody-biotin/epitope tag. This chain is not a pure dielectric; in fact, protein conductivity has been studied since the 1960s^35^. More recently, it has been shown that single peptide chains can support conductivities on the order of nanosiemens, corresponding to resistances in the hundreds of MΩ^36^. As we use deionized water in our electrode bath, this is likely the primary signal pathway that we isolate in our experiments (Fig. 4), and changes in the ionic strength of the water bath may affect our photoresponses. The QD material documentation references an overall diameter range of 27–34 nm for the particles; given that streptavidin and IgG molecules are 5–15 nm in size, and the biotin or peptide chains add another 1–5 nm, the total surface-surface distance in our experiments is on the order of 20 nm. This is substantially larger than a typical Debye distance of a conductive electrode immersed in an electrolyte bath of physiological salt concentrations (~1 nm), but much smaller than the Debye distance in pure deionized water (~1 μm). However, as the major intended purpose of the electrodes is to detect transient changes in local potential, a highly conductive DC channel may not be necessary, and electronic biosensors have been shown to detect robust signals at distances many times beyond the Debye distance^37^. Action potentials in the brain fire at frequencies of hundreds of Hz, with about 1 ms-long fronts of individual pulses, and have amplitudes of tens of mV, so our electrodes may well be able to detect such signals through AC coupling^38^ even if the interface ionic fluid cannot be fully displaced.

**Figure 5:**
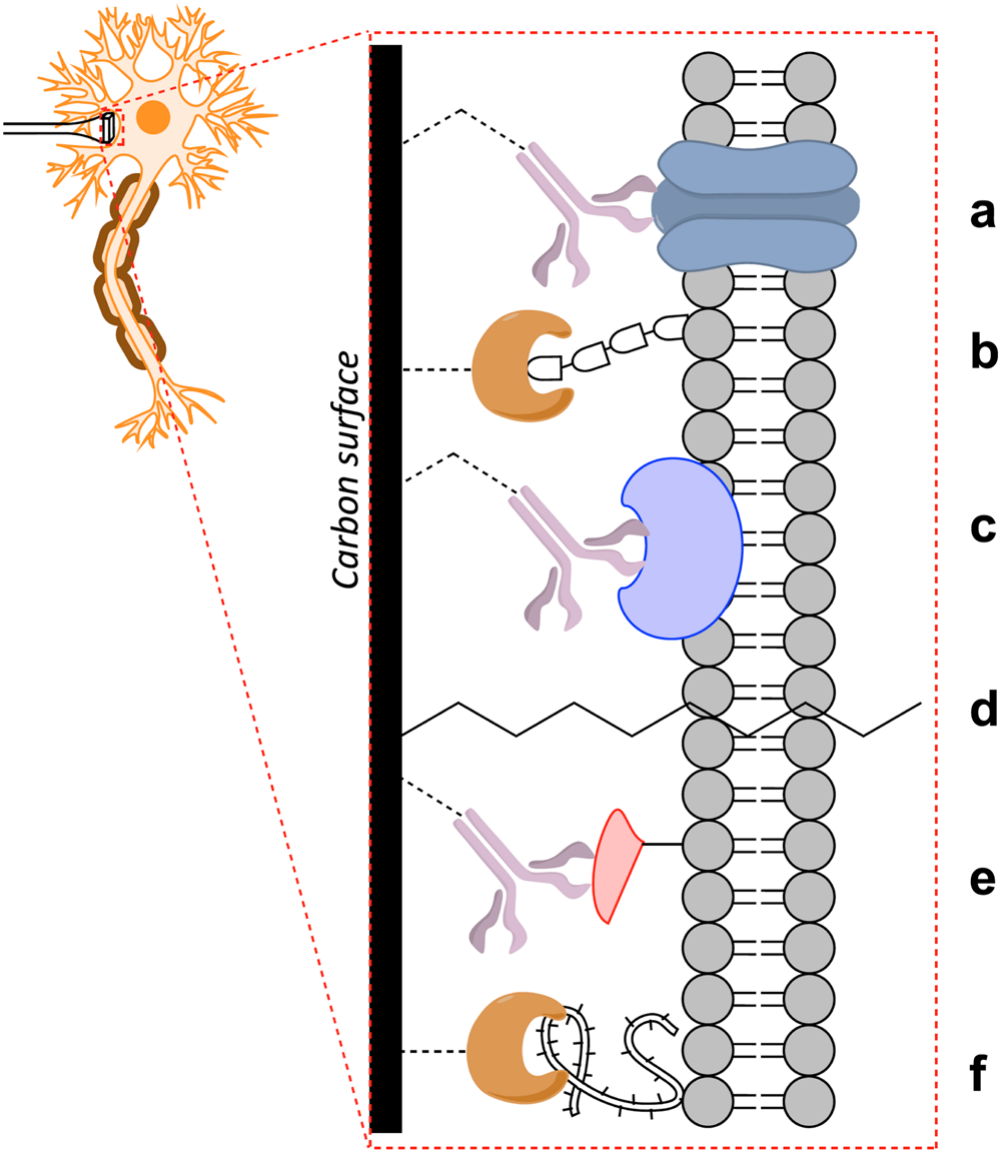
Proposed carbon surface modifications for direct neuronal interfaces. Common covalent modification strategies beyond SPPS could be used to simply and flexibly functionalize carbon electrodes (graphite, graphene, CNTs, carbon fibers, or other forms of carbon) for direct neuronal adhesion. Example attachments could include antibodies to transmembrane, intramembrane, and membrane-associated proteins (**a**,**c**,**e**), adhesive proteins to membrane phospholipid tails, glycoproteins, and ECM components (**b**,**f**), and lipids capable of adhering to and spanning the plasma membrane directly (**d**), among many others.

**Figure 6:**
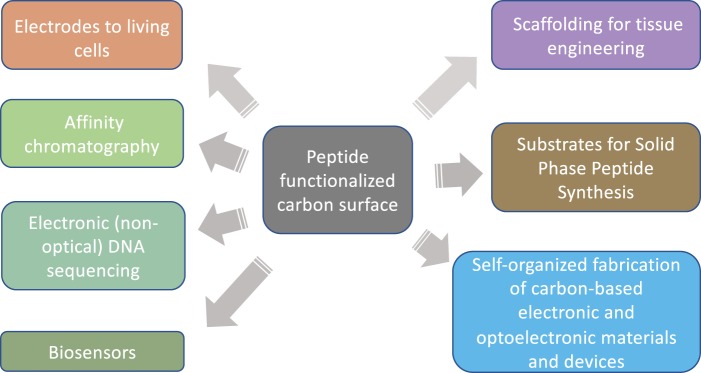
Proposed applications of peptide-functionalized carbon.

Straightforward organic reactivity of graphite and graphene opens these materials to an enormous range of secondary reactions and modifications. As shown in this work, TCNEO-modified graphite can act as a hub for de novo synthesis of polypeptides and be used as a resin for SPPS; it could also be similarly applied for other polymers. Common covalent modification strategies such as click chemistry could be recapitulated on graphite and graphene, and subsequently used to attach preformed peptides, proteins, lipids, nucleic acids, and other molecules of a range of complexities to these materials^39,40^. Photolithographic methods akin to those used in the manufacture of DNA microarrays could be applied for highly precise spatial patterning of such surfaces^41^. The demonstrated strong and specific affinity of functionalized surfaces to their corresponding binding molecules, in combination with previously published uses of similar materials in the purification of chiral compounds, builds a particularly intriguing body of evidence showcasing their efficacy and flexibility in chromatographic applications^23^. Otherwise, these materials could find use not only as electrodes for interactions with neurons but also in graphene-based DNA sequencing technologies, biomolecular sensors, and surfaces and scaffolds that display particular bioactive signals to interface with cultured or *in vivo* cells and specifically influence their behavior (Figs. 5, 6)^42–44^. Unlike prior attempts to apply graphene for such purposes, which predominantly used the processed forms of graphene oxide and reduced graphene oxide, our approach can be applied to pristine graphene, thus retaining the homogenous chemical structure of the material. Graphene also has a number of unique electronic properties, and while covalent modification is likely to significantly affect such characteristics in monolayer graphene, multi-layer forms of the material may retain at least some of them. Given the extensive recent progress in the industrial production of pristine graphene and the submicron shaping of this material using etching and photolithography^45^, the fabrication of precisely covalently functionalized carbon electrodes for highly specialized interactions with individual neurons and neuron cell membrane subdomains is imminent.

## Materials and Methods

### Materials and reagents

High-quality commercial polycrystalline graphite was obtained from SLG (SIGRAFINE R8650). Tetracyanoethylene oxide (TCNEO) (>97.0%, product T1439) was obtained from TCI America. Chlorobenzene (99.8%, product 284513), lithium aluminum hydride (1.0 M in diethyl ether, product 212792), tetrahydrofuran (THF) (≥99.9%, product 401757), 4-(dimethylamino)pyridine (DMAP) (≥99%, product 522805), N,N′-dicyclohexylcarbodiimide (DCC) (99%, product D80002), dichloromethane (≥99.8%, product 270997), N,N′-diisopropylcarbodiimide (DIC) (99%, product D125407), 1-hydroxybenzotriazole (HOBt) hydrate (97%, product 711489), piperazine (99%, product P45907), 1,8-diazabicyclo[5.4.0]undec-7-ene (DBU) (98%, product 139009), trifluoroacetic acid (TFA) (99%, product T6508), piperidine (99%, product 104094), triethylamine (≥99.5%, product 471283), and Fmoc chloride (97%, product 160512), as well as amino acid monomers Fmoc-Pro-OH (monomer P, ≥99.0%, product 47636), Fmoc-Tyr(tBu)-OH (monomer T, ≥98.0%, product 47623), Fmoc-Ala-OH (monomer A, 95%, product 531480), Fmoc-Gly-OH (monomer G, ≥98.0%, product 47627), Fmoc-Val-OH (monomer V, ≥98.0%, product 47638), and Fmoc-His(Trt)-OH (monomer H, ≥98.0%, product 47639), and bovine serum albumin (BSA) (≥96%, product A7888) and phosphate buffered saline (PBS) (powder, product P3813) were obtained from Sigma-Aldrich. D-(+)-biotin (product 2031) was obtained from Calbiochem. Fmoc-Asp(OMpe)-OH (monomer D, product 8.52104) was obtained from EMD Millipore. WesternDot 655 His-tag Mouse Monoclonal Antibody (product W10834), Qdot 585 Streptavidin Conjugate (product Q10111MP), and HA-tag Monoclonal Antibody (2–2.2.14) DyLight 550 (product 26183-D550), as well as normal mouse serum (product 10410) were obtained from Invitrogen.

### Graphite surface modification

#### TCNEO attachment and reduction

2 g of graphite discs and 40 mg of TCNEO were mixed in 7 mL chlorobenzene and refluxed at 150 °C for 5 days under atmospheric conditions. The mixture was also stirred from 24 hours after reaction start until its completion. The sample mixture was then filtered off, and the functionalized graphite was washed 3x with acetone, 3x with methanol, 3x with acetonitrile, and 3x with acetone. Sample **1** was dried at room temperature (RT) overnight (Fig. S6). Next, 1.0 g dry **1** was added to 3 mL dry THF. Under inert conditions (N_2_), 1 mL of 1 M LiAlH4 in diethyl ether was then added dropwise over 6 minutes. The reaction was stirred and proceeded at RT for 48 hours. After reaction completion, aqueous workup was performed, and the sample mixture was filtered off. The sample was neutralized to pH 7 and washed 3x with acetone, 3x with methanol, and 3x with acetone. Sample **2** was dried at RT overnight (Fig. S7).

#### Biotinylation of TCNEO-modified graphite

0.7 g dry **2**, 21.3 mg (8.73e-5 mmol) D-(+)-biotin, 24.0 mg (1.16e-4 mmol) DCC, and catalytic 5 mg (4.09e-5 mmol) DMAP were added to 3 mL dry dichloromethane under inert conditions (N_2_) and sonicated for 5 hours at RT. The sample was then washed 1x with deionized water, 3x with acetone, 3x with methanol, and 3x with acetone. Sample **3** was dried at RT overnight (Fig. S8).

#### Solid phase peptide synthesis on TCNEO-modified graphite

Solid phase peptide synthesis (SPPS) was implemented to attach His- and HA-tags to reduced TCNEO-modified graphite **2**. Fmoc-protected amino acid monomers, with tBu-, Trt- and OMpe-protected side chains, were used. Alternating steps of monomer attachment and Fmoc-deprotection were performed to build the peptide chains, followed by a final deprotection of side chain protecting groups. For the His-tag attachment, 10 H monomers were attached in a row. For the HA-tag, 13 monomers were attached in the order G-G-G-G-A-Y-D-P-V-D-Y-P-Y, with a 4-glycine linker connecting the epitope tag to the graphite surface (SPPS builds peptide chains in the C-terminus to N-terminus orientation) (Figs. S9, S10).

To begin with, 0.5 g dry **2** was added to 1 mL dry dichloromethane under inert conditions (Ar). In a separate flask, 55 mg HOBt and 50 mg of appropriate amino acid monomer were dissolved in 1 mL dry dichloromethane and 0.1–0.3 mL dry DMF (0.1 mL for H, 0.3 mL for all other monomers). The amino acid mixture was added to the sample flask along with 3.5 drops (5.5 equivalents to TCNEO) DIC, and the mixture was stirred for 4 hours at RT. The sample mixture was then filtered off, and the sample was washed 1x with deionized water, 3x with acetone, 3x with methanol, 3x with acetonitrile, and 3x with acetone. The sample **4/5** was dried at RT overnight.

For Fmoc-deprotection, a deprotection solution was made consisting of 8 mL dry DMF with 163.2 mg (2% w/v) DBU and 400 mg (5% w/v) piperazine. Dry sample was placed in 2 mL deprotection solution and shaken vigorously (900 rpm) at RT for 3–5 min (3 min for monomers 1–3 in the chain, 4 min for monomers 4–7, and 5 min for monomers 8–13). The solution was then decanted, another 2 mL was added to the sample, and it was again shaken vigorously (900 rpm) at RT for 12–14 more min (12 min for monomers 1–3, 13 min for monomers 4–7, and 14 min for monomers 8–13). Solution was again decanted, and the sample was then washed with 2 mL deprotection solution and 3x alternating washes of 2 mL dry DMF and 2 mL dry dichloromethane. The sample **6/7** was dried at RT overnight, and then proceeded to next monomer attachment in dichloromethane as above. These two steps were alternated until completion of peptide chains to samples **8/9**.

Final deprotection of His-tagged sample with Trt-group removal began with the addition of **8** to 3 mL dry dichloromethane with 44.7 mg (1%) TFA. This mixture was stirred at RT for 4 hours under inert conditions (N_2_). The sample mixture was then filtered off, and the sample was washed 3x with acetone, 3x with methanol, 3x with acetonitrile, and 3x with acetone. Sample **10** was dried overnight. Final deprotection of HA-tagged sample with tBu- and OMpe-group removal began with the addition of **9** to 3.85 mL (95%) TFA with 0.15 mL deionized water. This mixture was stirred at RT for 2 hours under inert conditions (Ar). The sample mixture was then filtered off, and the sample was washed 3x with acetone, 3x with methanol, 3x with acetonitrile, and 3x with acetone. Sample **11** was dried overnight.

### Binding protein attachment to functionalized graphite

Two buffer solutions were prepared for binding protein attachment experiments: blocking buffer (6% BSA/10% normal mouse serum in 1X PBS) and incubation buffer (6% BSA in 1X PBS). All binding protein stocks were centrifuged at 5000 g for 3 min prior to use, and aliquots were taken from the supernatant. Binding proteins were diluted in incubation buffer at ratio 1:2 for HA-tag Monoclonal Antibody (2–2.2.14) DyLight 550 and 1:10 for WesternDot 655 His-tag Mouse Monoclonal Antibody and Qdot 585 Streptavidin Conjugate. Surface-modified graphite samples were incubated in blocking buffer for 1 hour, then with binding proteins in incubation buffer for 1 hour. Afterwards, they were washed 3x with 1X PBS for 5 min each and kept in 1X PBS for spectroscopic and photoresponse measurements. All steps were performed at RT.

### Spectroscopic methods and instruments

Auger spectroscopy was performed using a Perkin-Elmer PHI 600 Scanning Auger Multiprobe with RBD Digital Acquisition. Measurements were performed using a 3 keV beam over an area of 200 × 200 µm, with samples tilted at 30° off the electron beam normal toward the ion gun. Raman spectroscopy was performed using a Horiba Jobin Yvon Aramis micro-Raman spectrometer with cooled Jobin Yvon Synapse CCD detector. A frequency-doubled diode-pumped Nd:YAG laser was used for excitation (emission wavelength 532 nm, radiation power 5 mW, CW regime, spot size 15 µm). SEM and EDX analyses were performed using a Hitachi S-3200N scanning electron microscope with Thermo Electron LN-Cooled EDX detector with spectral resolution 134 eV. Electron beam energy was 10 keV, and EDX spectra were collected from 3000 µm^2^ area of sample surface.

### Photoresponse methods and instruments

The circuit setup for the photoresponse measurements used an Ametec 5113 voltage amplifier and a Tektronix TDS3052B oscilloscope. A quartz-halogen lamp was used for broad-spectrum illumination, providing 100 mW power over an area of 4 mm diameter. Laser illumination sources included a diode-pumped second harmonic YAG laser emitting at 532 nm with 5 mW power, a HeNe laser emitting at 633 nm with 15 mW power, and a semiconductor laser emitting at 785 nm with 10 mW power. Laser sources were intentionally defocused to a spot diameter of 2 mm.

### Data analysis and visualization

Data analysis was performed in Origin Pro 9. Chemical structures were drawn using ChemDraw Professional 17.1. All schematics were created in Adobe Illustrator CC 22.1 and ChemDraw Professional 17.1 using included copyright-free templates.

## Supplementary information

Supplementary Information

## Data Availability

All data needed to evaluate the conclusions in the paper are present in the paper and/or the Supplementary Information.

## References

[1] Hodgkin AL, Huxley AF (1939). Action potentials recorded from inside a nerve fibre. Nature.

[2] Polikov VS, Tresco PA, Reichert WM (2005). Response of brain tissue to chronically implanted neural electrodes. Journal of Neuroscience Methods.

[3] Biran R, Martin DC, Tresco PA (2005). Neuronal cell loss accompanies the brain tissue response to chronically implanted silicon microelectrode arrays. Experimental Neurology.

[4] Stieglitz T (2004). Considerations on surface and structural biocompatibility as prerequisite for long-term stability of neural prostheses. J. Nanosci. Nanotechnol..

[5] Cohen AE, Venkatachalam V (2014). Bringing bioelectricity to light. Annual Review of Biophysics.

[6] Obien MEJ, Deligkaris K, Bullmann T, Bakkum DJ, Frey U (2015). Revealing neuronal function through microelectrode array recordings. Front. Neurosci..

[7] Spira ME, Hai A (2013). Multi-electrode array technologies for neuroscience and cardiology. Nature Nanotechnology.

[8] Kruskal PB, Jiang Z, Gao T, Lieber CM (2015). Beyond the Patch Clamp: Nanotechnologies for Intracellular Recording. Neuron.

[9] Rand, D. & Hanein, Y. Carbon nanotubes for neuron–electrode interface with improved mechanical Performance, In *Nanotechnology and Neuroscience: Nano-electronic, Photonic and Mechanical Neuronal Interfacing*, De Vittorio, M. *et al*., Eds. (Springer Science+Business Media, New York, 2014).

[10] Aqrawe Z, Montgomery J, Travas-Sejdic J, Svirskis D (2018). Conducting polymers for neuronal microelectrode array recording and stimulation. Sensors and Actuators B: Chemical.

[11] Rivnay J, Owens RM, Malliaras GG (2014). The rise of organic bioelectronics. Chem. Mater..

[12] Torsi L, Magliulo M, Manolia K, Palazzo G (2013). Organic field-effect transistor sensors: a tutorial review. Chem. Soc. Rev..

[13] Głowacki ED (2013). Hydrogen-bonded semiconducting pigments for air-stable field-effect transistors. Adv Mater..

[14] Liu J (2015). Syringe-injectable electronics. Nat. Nanotechnol..

[15] Hong G, Yang X, Zhou T, Lieber CM (2018). Mesh electronics: a new paradigm for tissue-like brain probes. Current Opinion in Neurobiology.

[16] Hong G (2018). A method for single-neuron chronic recording from the retina in awake mice. Science.

[17] Seabra AB, Paula AJ, de Lima R, Alves OL, Durán N (2014). Nanotoxicity of graphene and graphene oxide. Chem. Res. Toxicol..

[18] Fabbro A (2016). Graphene-based interfaces do not alter target nerve cells. ACS Nano.

[19] Vomero M, Oliveira A, Ashouri D, Eickenscheidt M, Stieglitz T (2018). Graphitic carbon electrodes on flexible substrate for neural applications entirely fabricated using infrared nanosecond laser technology. Scientific Reports.

[20] Bekyarova E, Sarkar S, Niyogi S, Itkis ME, Haddon ME (2012). Advances in the chemical modification of epitaxial graphene. J Phys D: Appl Phys.

[21] Magedov IV (2013). Benzyne-functionalized graphene and graphite characterized by Raman spectroscopy and energy dispersive X-ray analysis. Carbon.

[22] Frolova LV (2015). Tetracyanoethylene oxide-functionalized graphene and graphite characterized by Raman and Auger spectroscopy. Carbon.

[23] Candelaria L (2018). Surface-modified three-dimensional graphene nanosheets as a stationary phase for chromatographic separation of chiral drugs. Scientific Reports.

[24] Jarvik JW, Telmer CA (1998). Epitope tagging. Annual Review of Genetics.

[25] Merrifield RB (1963). Solid phase peptide synthesis. I. The synthesis of a tetrapeptide. J. Am. Chem. Soc..

[26] *Methods of surface analysis*, Wolsky, S. P. & Czanderna, A. W. Eds., (Elsevier, Amsterdam, 1988), Elsevier e-book, (2012).

[27] Ferrari AC (2007). Raman spectroscopy of graphene and graphite: Disorder, electron phonon coupling, doping and nonadiabatic effects. Sol. St. Comm..

[28] Kim HY (2016). Quantum dot/siloxane composite film exceptionally stable against oxidation under heat and moisture. Journal of the American Chemical Society.

[29] Pokrant S, Whaley KB (1999). Tight-binding studies of surface effects on electronic structure of CdSe nanocrystals: the role of organic ligands, surface reconstruction, and inorganic capping shells. Eur. Phys. J. D.

[30] Głowacki ED (2015). Bioconjugation of hydrogen-bonded organic semiconductors with functional proteins. J. Mater. Chem. C.

[31] Smith AM, Lane LA, Nie S (2014). Mapping the spatial distribution of charge carriers in quantum-confined heterostructures. Nature Communications.

[32] Pampaloni NP (2018). Single-layer graphene modulates neuronal communication and augments membrane ion currents. Nature Nanotechnology.

[33] Bramini M (2018). Interfacing graphene-based materials with neural cells. Front. Syst. Neurosci..

[34] Fadeel B (2018). Safety assessment of graphene-based materials: focus on human health and the environment. ACS Nano.

[35] Rosenberg B (1962). Electrical Conductivity of Proteins. Nature.

[36] Zhang B (2019). Role of contacts in long-range protein conductance. PNAS.

[37] Palazzo G (2015). Detection beyond Debye’s length with an electrolyte-gated organic field-effect transistor. Advanced Materials.

[38] Tian B (2018). Roadmap on semiconductor–cell biointerfaces. Physical Biology.

[39] Yi G, Son J, Yoo J, Park C, Koo H (2018). Application of click chemistry in nanoparticle modification and its targeted delivery. Biomater Res..

[40] Ahmad Fuaad AA, Azmi F, Skwarczynski M, Toth I (2013). Peptide conjugation via CuAAC ‘click’ chemistry. Molecules.

[41] Fodor SP (1991). Light-directed, spatially addressable parallel chemical synthesis. Science.

[42] Heerema SJ, Dekker C (2016). Graphene nanodevices for DNA sequencing. Nature Nanotechnology.

[43] Peña-Bahamonde J, Nguyen HN, Fanourakis SK, Rodrigues DF (2018). Recent advances in graphene-based biosensor technology with applications in life sciences. Journal of Nanobiotechnology.

[44] Priyadarsini S, Mohanty S, Mukherjee S, Basu S, Mishra M (2018). Graphene and graphene oxide as nanomaterials for medicine and biology application. Journal of Nanostructure in Chemistry.

[45] Bonaccorso F (2012). Production and processing of graphene and 2D crystals. Materials Today.

