# Supplementary material for: Covalent Epitope Decoration of Carbon Electrodes using Solid Phase Peptide Synthesis
Source: Sci Rep. 2019 Nov 28;9:17805. doi: 10.1038/s41598-019-54000-9 (PMC6882871; doi:10.1038/s41598-019-54000-9)
Supplement: Supplementary file 1 — Supplementary Information [file 41598_2019_54000_MOESM1_ESM.pdf]

Supplementary Information for

Covalent Epitope Decoration of Carbon Electrodes using Solid Phase Peptide Synthesis

Lindsay Candelaria, Peter N. Kalugin, Brian M. Kowalski, Nikolai G. Kalugin*

*Corresponding author: nikolai.kalugin@nmt.edu

The PDF file includes:

Fig. S1. Auger spectra of graphite surfaces at various stages of modification.
Fig. S2. Raman spectra of graphite surfaces at various stages of modification.
Fig. S3. EDX spectra of graphite surfaces at various stages of modification.
Fig. S4. SEM images of graphite surfaces with superimposed EDX spectra.
Fig. S5. Reproducible detection of photoinduced electrical potentials near quantum dot surfaces and chemical fluorophores.
Fig. S6. TCNEO modification of graphite.
Fig. S7. Reduction of TCNEO-modified graphite.
Fig. S8. Biotinylation of TCNEO-modified graphite.
Fig. S9. SPPS of TCNEO-modified graphite: His-tag attachment.
Fig. S10. SPPS of TCNEO-modified graphite: HA-tag attachment.

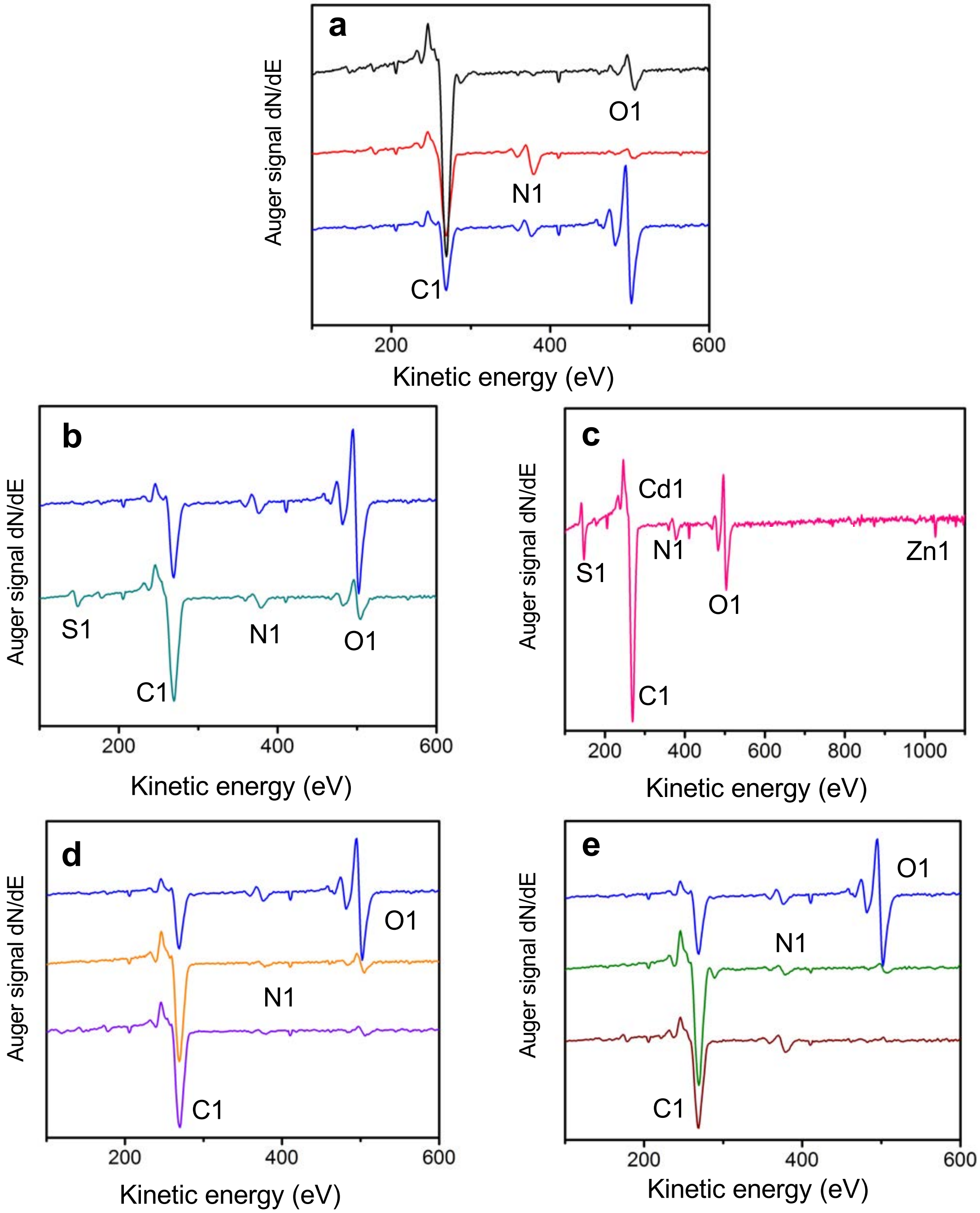

Fig. S1. Auger spectra of graphite surfaces at various stages of modification. (**a**) Auger spectra of unmodified (black), TCNEO-modified (red), and reduced TCNEO-modified (blue) graphite. (**b**) Auger spectra of reduced TCNEO-modified (blue) and biotinylated (teal) graphite. (**c**) Auger spectrum of biotinylated graphite with associated streptavidin-coated quantum dots. (**d**) Auger spectra of reduced TCNEO-modified (blue), His-tag-functionalized (gold), and His-tag-functionalized with associated His-tag antibody-coated quantum dots (purple) graphite. (**e**) Auger spectra of reduced TCNEO-modified (blue), HA-tag-functionalized (green), and HA-tag-functionalized with associated fluorophore-coated HA-tag antibodies (maroon) graphite. Relevant peaks are labeled.

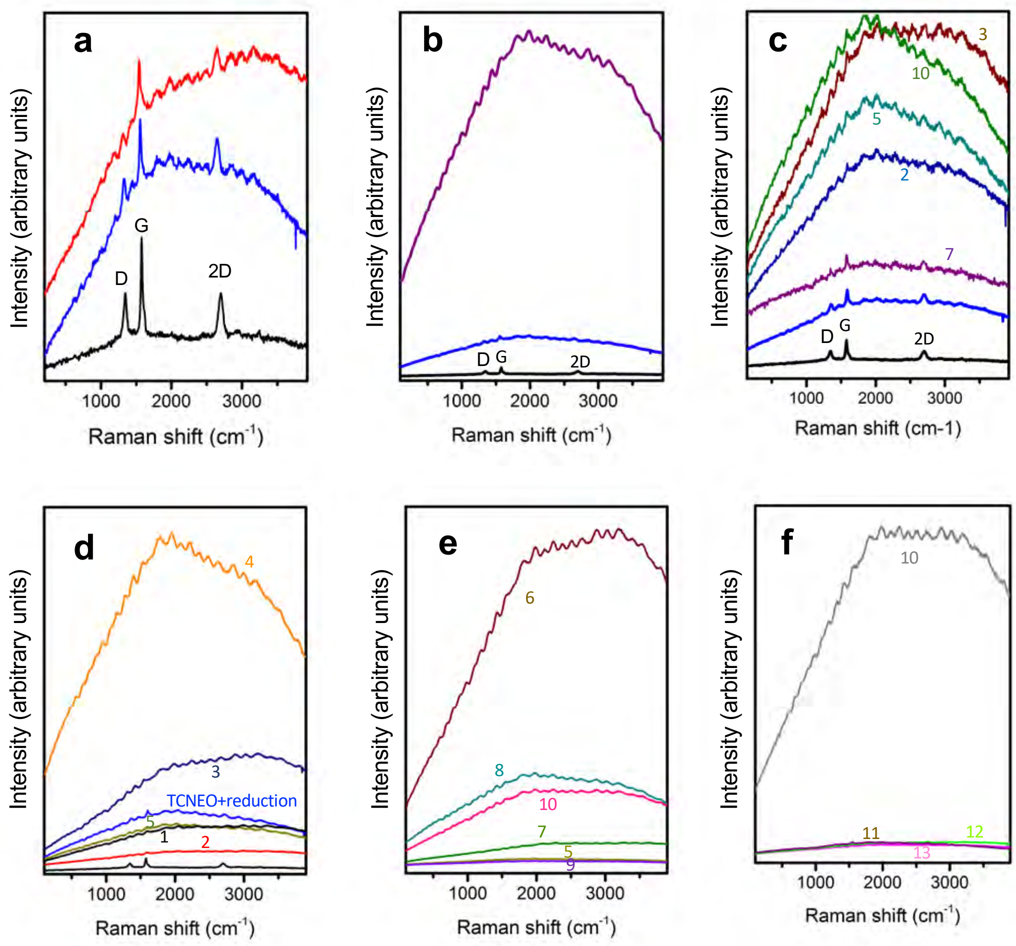

Fig. S2. Raman spectra of graphite surfaces at various stages of modification. (**a**) Raman spectra of unmodified (black), TCNEO-modified (red), and reduced TCNEO-modified (blue) graphite. (**b**) Raman spectra of unmodified (black), reduced TCNEO-modified (blue), and biotinylated (purple) graphite. (**c**) Raman spectra of unmodified (black), reduced TCNEO-modified (light blue), and polyhistidine-functionalized graphite at various stages of His-tag assembly by SPPS. Number reflects length of attached polyhistidine peptide to a full length of 10 amino acids. (**d**, **e**, **f**) Raman spectra of unmodified (black), reduced TCNEO-modified (light blue, labeled), and polypeptide-functionalized graphite at various stages of HA-tag assembly by SPPS. Number reflects length of attached polypeptide to a full length of 13 amino acids. Excitation wavelength 532 nm for all measurements, relevant peaks are labeled.

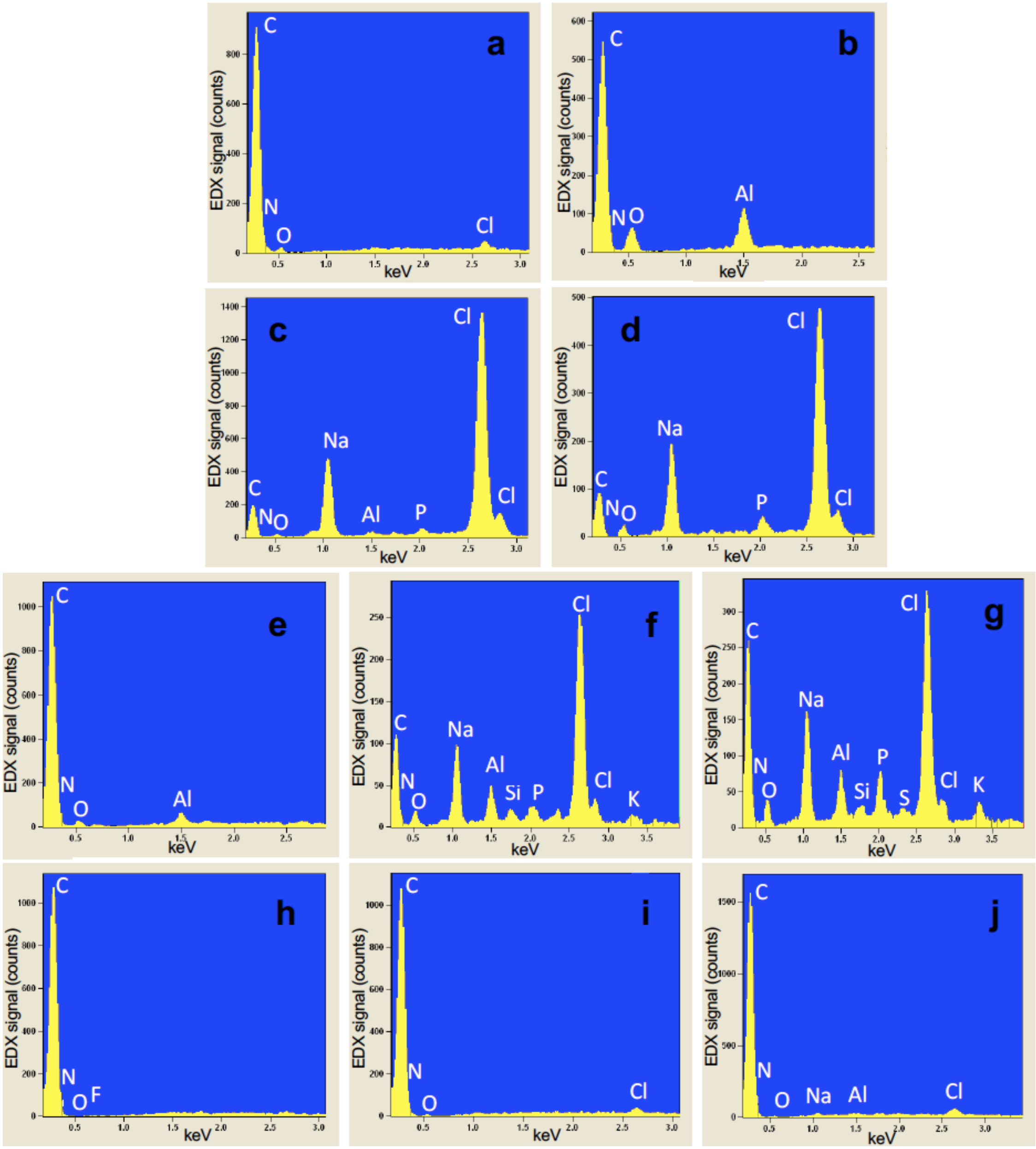

Fig. S3. EDX spectra of graphite surfaces at various stages of modification. (**a**) TCNEO-modified, (**b**) reduced TCNEO-modified, (**c**) biotinylated and treated with streptavidin-coated quantum dots, (**d**) biotinylated and treated with His-tag antibody-coated quantum dots, (**e**) His-tag-functionalized, (**f**) His-tag-functionalized and treated with His-tag antibody-coated quantum dots, (**g**) His-tag-functionalized and treated with streptavidin-coated quantum dots, (**h**) HA-tag-functionalized, (**i**) HA-tag-functionalized and treated with fluorophore-coated HA-tag antibodies, (**j**) HA-tag-functionalized and treated with His-tag antibody-coated quantum dots. Electron beam energy 10 keV for all measurements, collection area 3000 μm^2, relevant peaks are labeled.

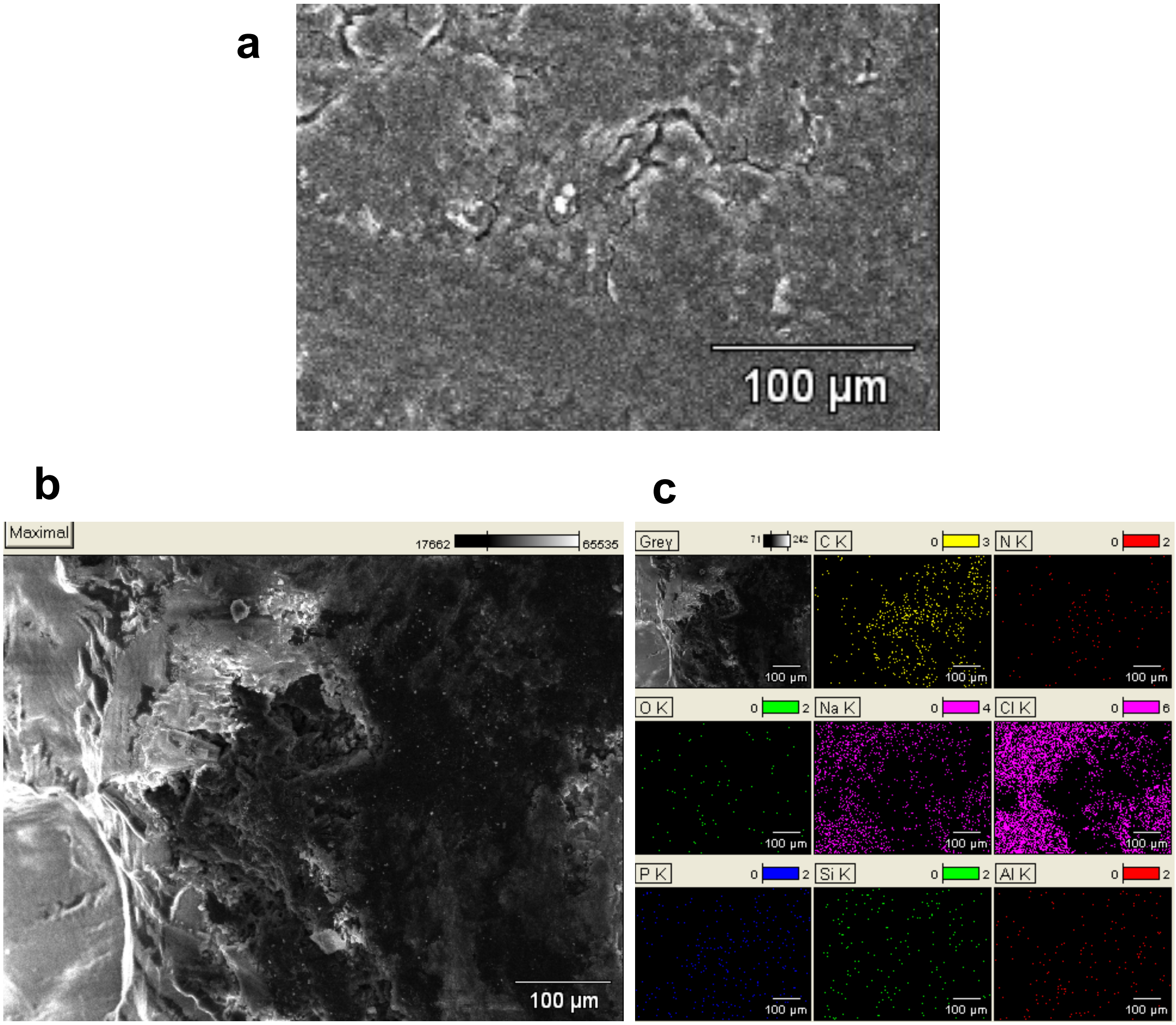

Fig. S4. SEM images of graphite surfaces with superimposed EDX spectra. (**a**) SEM image of TCNEO-modified graphite. (**b**) SEM image of biotinylated graphite (selected area with a network of graphite cleavage-generated defects) with attached streptavidin-coated quantum dots. (**c**) EDX intensity map of the area in (**b**), showing the spatial distribution of signals corresponding to C, N, O, Na, Cl, P, Si, and Al, as labeled. Electron beam energy 10 keV for all measurements, collection area 3000 μm^2.

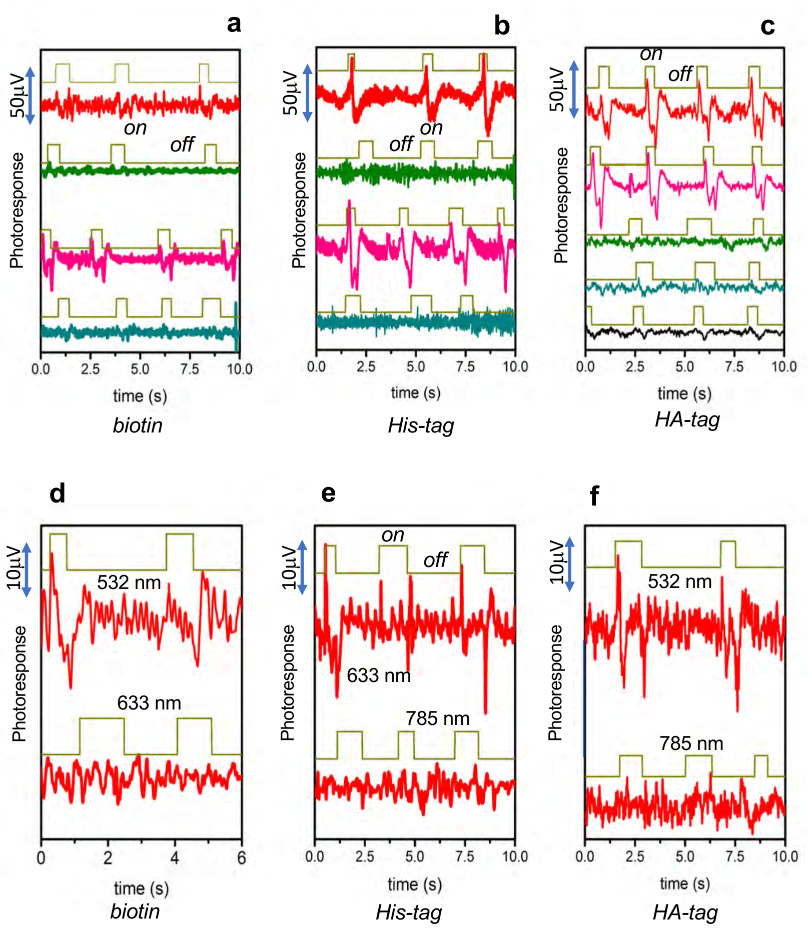

Fig. S5. Reproducible detection of photoinduced electrical potentials near quantum dot surfaces and chemical fluorophores. Photoinduced electrical potential traces were obtained with irradiation by a broad-spectrum quartz-halogen lamp, with each trace corresponding to independently fabricated samples of (**a**) biotin-functionalized graphite incubated with streptavidin-coated quantum dots (red and pink) and His-tag antibody-coated quantum dots (olive and teal), (**b**) His-tag-functionalized graphite incubated with His-tag antibody-coated quantum dots (red and pink) and streptavidin-coated quantum dots (olive and teal), and (**c**) HA-tag-functionalized graphite incubated with buffer only (black), fluorophore-coated HA-tag antibodies (red and pink), and His-tag antibody-coated quantum dots (olive and teal). Photoinduced electrical potential traces were also obtained with wavelength-specific laser irradiation. (**d**) Biotin-functionalized graphite incubated with streptavidin-coated quantum dots (585 nm emission) was irradiated with lasers emitting at 532 nm (top) and 633 nm (bottom). (**e**) His-tag-functionalized graphite incubated with His-tag antibody-coated quantum dots (655 nm emission) was irradiated with lasers emitting at 633 nm (top) and 785 nm (bottom). (**f**) HA-tag-functionalized graphite incubated with fluorophore-coated HA-tag antibodies (576 nm emission) was irradiated with lasers emitting at 532 nm (top) and 785 nm (bottom). Excitation wavelength dependence is notable in all cases, with laser illumination of energy above the emission wavelength of a given fluorophore capable of stimulating a potential difference, while lower energy excitation gives no noticeable signal. The type of graphite functionalization is indicated in *italics*.

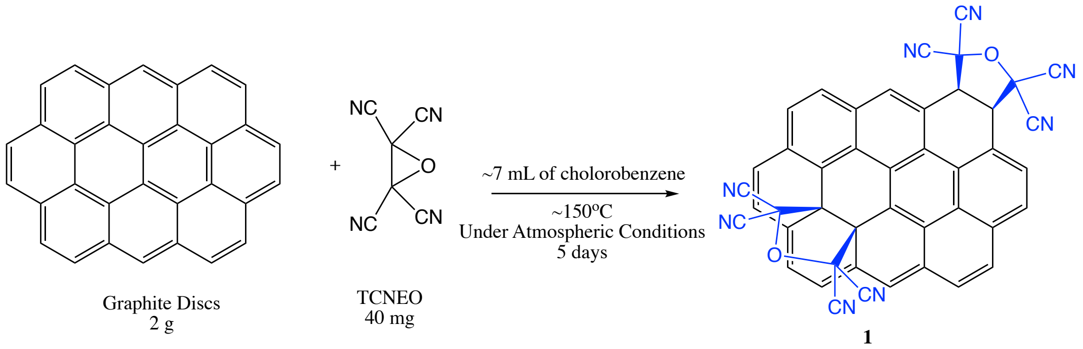

Fig. S6. TCNEO modification of graphite.

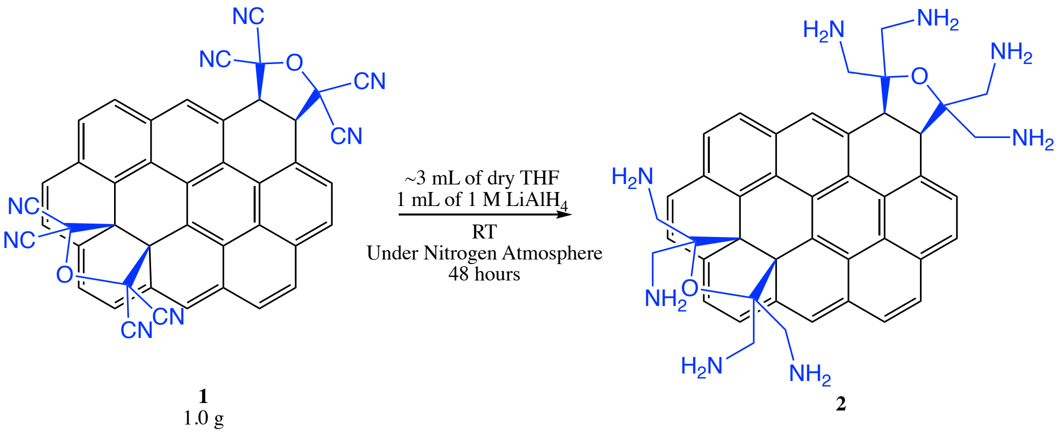

Fig. S7. Reduction of TCNEO-modified graphite.

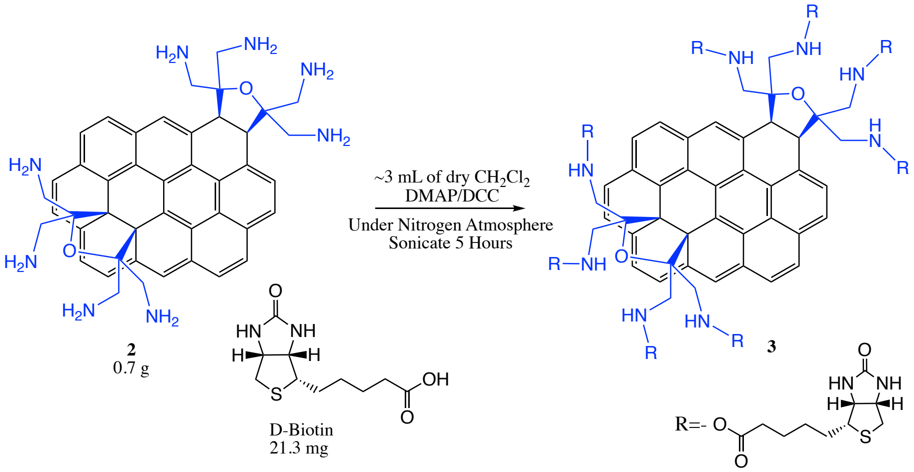

Fig. S8. Biotinylation of TCNEO-modified graphite.

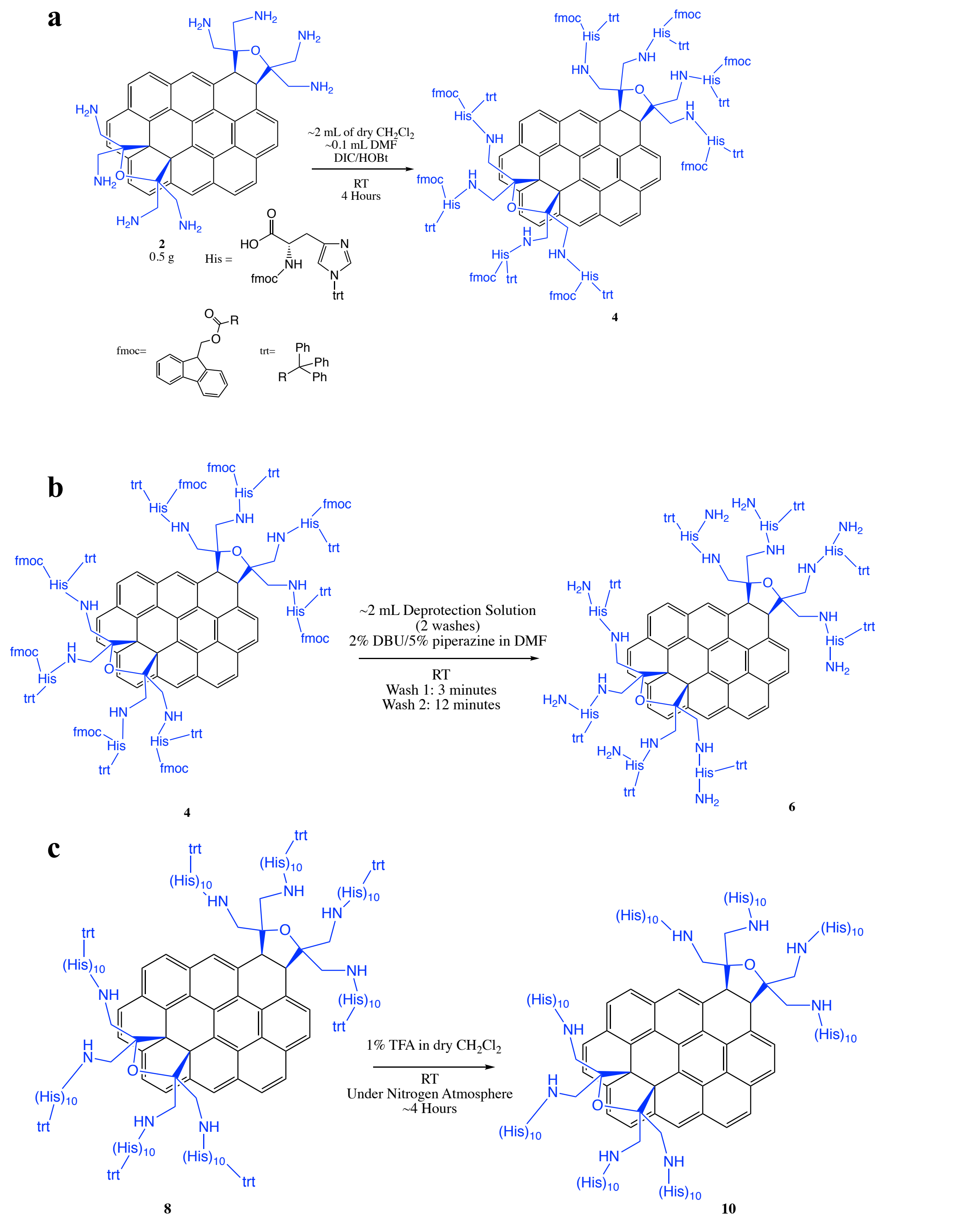

Fig. S9. SPPS of TCNEO-modified graphite: His-tag attachment. (**a**) Histidine monomer attachment. (**b**) Fmoc deprotection of terminal monomer. (**c**) Final side chain deprotection.

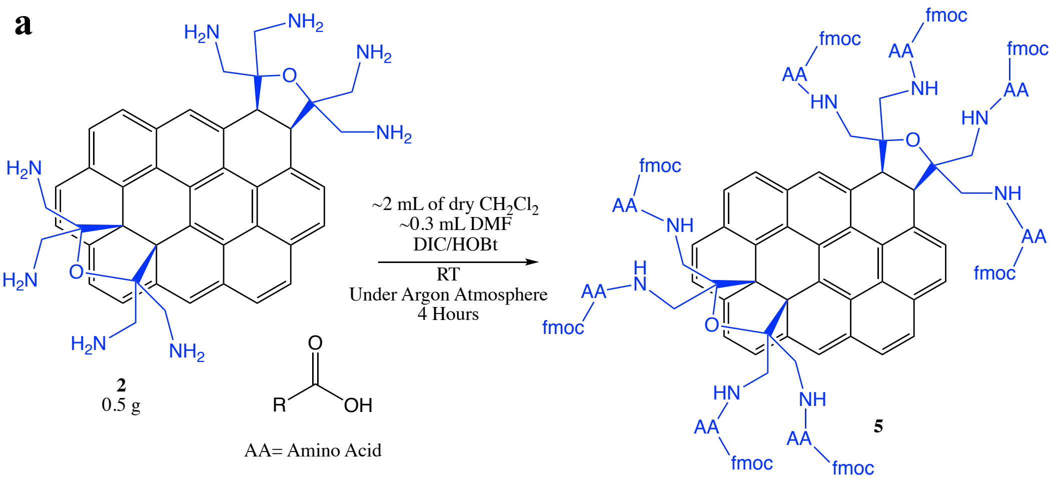

-Synthesis (coupling) of amino acids was specific to Ha-tag sequence
-Aspartic Acid residues contained OMpe protecting groups
-Tyrosine residues contain –tBu protecting groups
-Glycine (4) residues were added initially to extend overall length of the available Ha-tag chain

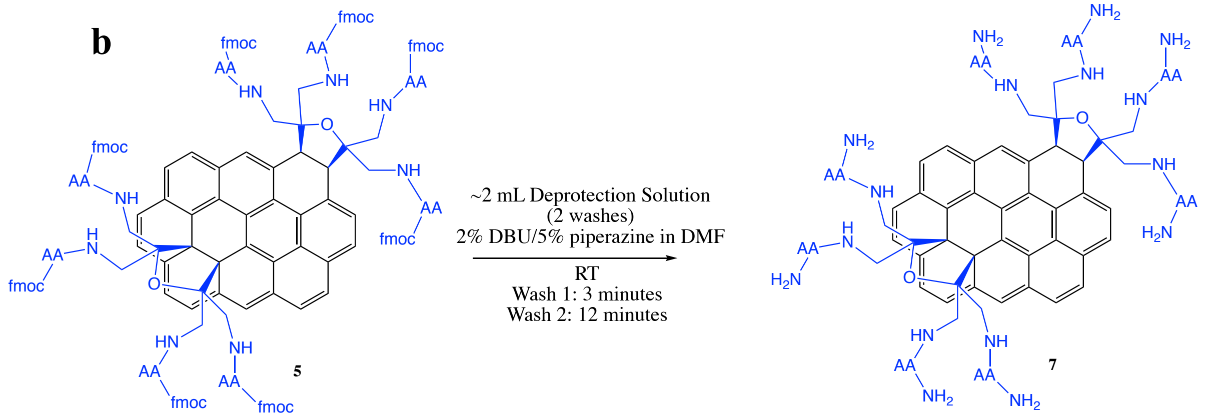

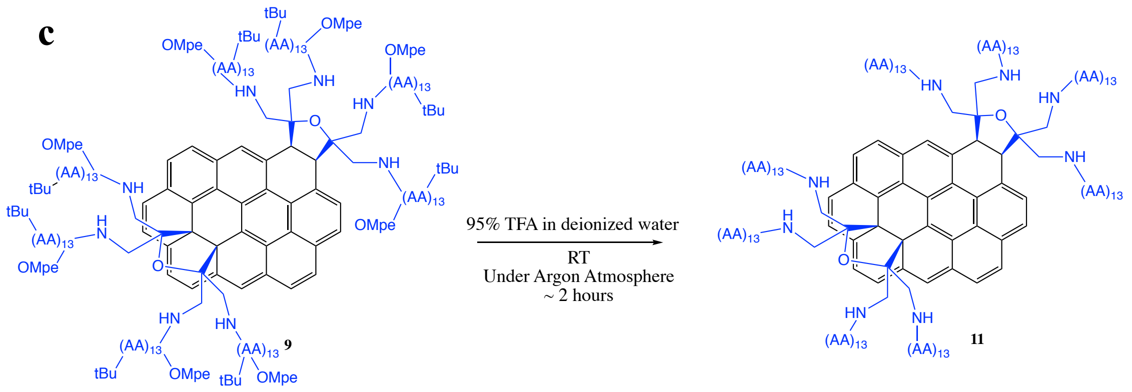

AA-Amino Acid

Fig. S10. SPPS of TCNEO-modified graphite: HA-tag attachment. (**a**) Amino acid monomer attachment. (**b**) Fmoc deprotection of terminal monomer. (**c**) Final side chain deprotection.
